# Facilitating Screening of MOFs for Mixed Matrix Membranes Using Machine Learning and the Maxwell Model

**DOI:** 10.1021/acs.jpcc.5c01483

**Published:** 2025-05-01

**Authors:** Xiaohan Yu, Jia Yuan Chng, David S. Sholl

**Affiliations:** † School of Chemical & Biomolecular Engineering, Georgia Institute of Technology, Atlanta, Georgia 30332-0100, United States; ‡ Oak Ridge National Laboratory, Oak Ridge, Tennessee 37830, United States

## Abstract

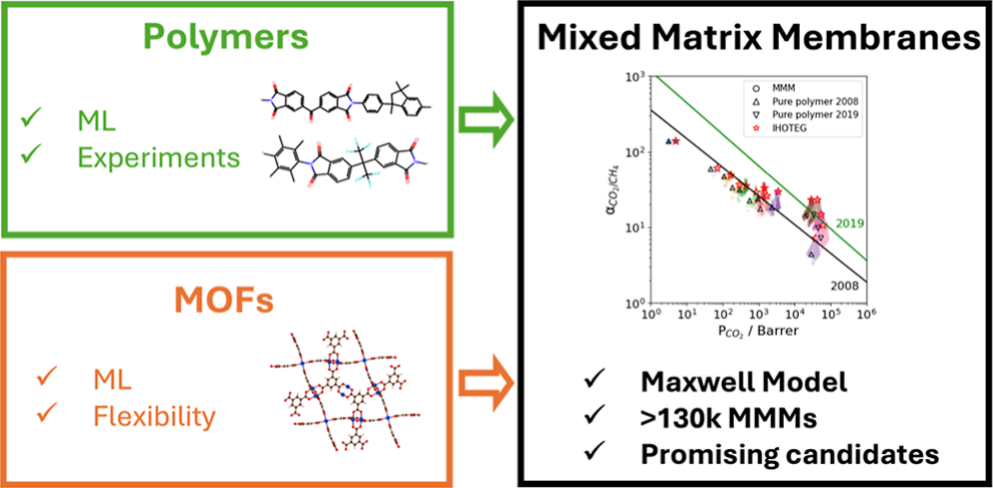

Metal organic framework (MOF)-based mixed-matrix membranes (MMMs), which embed MOF particles in polymer matrices, combine the advantages of polymeric and inorganic membranes. Multiple previous studies have used the Maxwell model together with molecular simulations and machine learning (ML) to predict the performance of MOF/polymer MMMs. However, the assumption of rigid MOF frameworks in molecular simulations limited the accuracy of the data used in the predictions, particularly in predicting molecular diffusivities. We developed a novel workflow integrating ML models with consideration of MOF flexibility to predict the permeability and selectivity of 131,722 MMMs for CO_2_/CH_4_, O_2_/N_2_ and He/H_2_ separations. The full range of achievable MMM performance within the Maxwell model was analyzed, and several promising MOFs were identified using this workflow. This approach offers an efficient tool for screening any polymer and MOF combination in gas separation applications.

## 1 Introduction

Separation processes are essential across a broad range of industrial applications but are often highly energy intensive. Distillation as the most dominant separation process takes up 10–15% of the world’s energy consumption by some estimates.^1^ Gas separations using polymer-based membranes are a promising alternative that significantly reduces the required energy and capital costs.^2,3^ However, conventional polymeric membranes suffer from a trade-off between selectivity and gas permeability, as represented by the Robeson upper bound.^4^ Other challenges associated with polymeric membranes include aging,^5^ plasticization,^6^ and limited thermal stability.^7^

Nonpolymeric membranes, including metal–organic framework (MOF) membranes, have been developed in part to address some of the intrinsic limitations of polymeric membranes. MOFs are a diverse class of crystalline materials that have been widely explored in separations due to their high porosity, high surface area and high tunability.^8^ Many pure MOF membranes show both excellent permeability and selectivity.^9−12^ Nevertheless, it remains challenging to fabricate MOF membranes with consistent properties while maintaining their integrity on the scales needed for industrial separations.^13^

A widely explored approach to combine positive features of polymeric and inorganic membranes is to incorporate inorganic particles into polymer matrices creating mixed-matrix membranes (MMMs). In MMMs based on MOFs the polymer matrix is a continuous phase while MOF particles as the filler phase are dispersed in the polymer matrix. Multiple successful MMMs with MOF fillers have been synthesized in experimental studies.^11^ For separations such as CO_2_/CH_4_,^14−16^ H_2_/CH_4_,^15^ and C_3_H_6_/C_3_H_8_,^17−19^ MMMs have been reported with permselectivity surpassing the Robeson upper bound for polymer membranes. It has been argued that MMMs offer one avenue to significantly improve the efficiency and productivity of current membrane-based separations.^3^

One appealing feature of MMMs is the enormous diversity of polymer/filler combinations that can be considered for each separation of interest. Thousands of MOFs and thousands of polymers have been synthesized,^20,21^ so millions of polymer/MOF combinations exist. For obvious practical reasons, however, only a miniscule fraction of these combinations has been studied experimentally. Since at least 2010 this observation has motivated efforts to use models that combine experimental data and data from molecular simulations to make predictions about polymer/MOF MMMs.^22^

In recent years efforts have been made with machine learning (ML) methods to predict MMM permeabilities and selectivities from either experimental or simulation data. Guan et al. collected 648 experimental data points on MMMs (including 36 MOFs and 41 polymers) for CO_2_/CH_4_ separation and trained a random forest ML model to accurately predict direct MMM permeability and selectivity.^23^ They successfully synthesized MMMs with MOFs predicted to perform well and the resulting MMMs surpassed the Robeson upper bound. This ML model was also successfully transferred to make predictions about CO_2_/N_2_ separations. Alizamir et al. used alternate ML methods based on the data set collected by Guan et al.^24^ Similarly, Yao et al. predicted CO_2_/N_2_ separation performance based on 291 MMM experimental data points including 43 types of MOFs and 19 types of polymers.^25^ Despite the success of this approach, it is limited by the amount of available experimental data for MMMs, and it is far from clear that the extant experimental data allows reliable extrapolation to the full diversity of polymer and MOF materials that exist.

An alternative approach to predict the performance of MMMs is to separately predict the properties of polymers and fillers. The most common basis for these predictions, and the one we will use in this paper, is the Maxwell model, which offers the simplest prediction of permeance through a MMM based on properties of the individual phases.^26−31^ The Maxwell model ignores any effects due to particle size or preferential orientation of the filler particles, and various approaches that incorporate these effects have been explored.^32−36^ Perhaps more importantly, the Maxwell model assumes that the interfaces between the matrix and filler are ideal in the sense that they do not change the permselectivity of either phase. It is widely accepted that this assumption is, at best, only approximate and that truly quantitative models of MMMs should incorporate information about these interfaces. Carefully characterizing these interfaces experimentally or making meaningful predictions about them with simulations, however, remains very challenging even for individual polymer/MOF pairs, and it is certainly not something that can currently be achieved in any high throughput sense.^37−40^ In this paper, we therefore focus on the challenge of making accurate predictions of the performance of MMMs in the limit in which the Maxwell model is valid.

Multiple studies have reported predictions for large collections of polymer/MOF MMMs using a combination of molecular simulation data and/or ML methods.^26−31,35,41−47^ To evaluate the generality and accuracy of these models it is important to consider the sources of data that were used for polymer properties and MOF properties. Most existing studies have used experimental data for the permeability of polymers.^26−31,35,41−47^ This choice has the advantage that the resulting predictions are based in physical reality, but typically makes it difficult to extend the results to the large numbers of alternative polymers that exist but have not been studied in previous experiments. Fortunately, several powerful ML models predicting gas permeance in polymers have now been developed,^48−52^ creating an opportunity to make the input into MMM models regarding the polymer phase far more generalizable.

In contrast to the polymer phase, predictions of MOF properties for use in the Maxwell model have relied almost exclusively on molecular simulations.^26−30,35,41−47^ Using simulation data in this way requires separate predictions for the adsorption properties of the MOF and the diffusivities of adsorbed molecules, information that can then be combined through the solution–diffusion model to predict permeance.^53^ A number of studies have tackled the challenge of using ML for predicting adsorption isotherms for molecules in MOFs based on molecular simulation data. Most of these studies have focused on predictions for a specific molecule or a small number of molecules.^54−62^ We have recently developed a more general ML approach that applies to a very wide range of molecules in MOFs of arbitrary composition and structure.^63^

Although the accuracy of both adsorption and diffusion data for the MOF phase is desirable, the accuracy of predictions about molecular diffusivities is arguably particularly important because diffusivities can vary across orders of magnitude as pore sizes and characteristics of nanoporous crystals are varied. A strong limitation in most molecular simulations of diffusion in MOFs, especially high throughput studies, is that for reasons of computational efficiency they approximate the MOF as being rigid. There are numerous examples where comparisons with more physically accurate simulations based on flexible MOF structures where simulations based on rigid MOFs underestimated molecular diffusivities by orders of magnitude.^64−69^ This observation means that previous models of MMMs that relied of MOF diffusion simulation data from rigid MOFs, while illustrating the potential scope for screening large numbers of MMMs, are in many cases based on highly inaccurate underlying data.

Yang and Sholl curated the largest data set available to date with diffusion information from molecular simulations that include framework flexibility in MOFs.^70^ For rigid molecules, many cases showed that simulations using rigid frameworks underestimate diffusivity in fully flexible frameworks by several orders of magnitude, particularly in MOFs with relatively small pores. Interestingly, Yang and Sholl also identified many examples of large-pore MOFs where the diffusivity of flexible molecules was greatly overestimated by simulations assuming rigid frameworks. Unfortunately, the quantity of data available from these simulations is too small to develop reliable ML-based predictions of molecular diffusivities in arbitrary MOFs that includes information about MOF flexibility. Crucially, however, Yang et al. developed a robust ML classifier to predict whether MOF flexibility influences the self-diffusion coefficient of adsorbates, along with a regressor based on large-scale prior simulations with rigid structures to predict MOF diffusion coefficients in cases where flexibility has a negligible impact.^71^ Restricting attention to the latter class of materials means that a large source of potential inaccuracy in earlier models of polymer/MOF MMMs can be avoided.

In this paper, we demonstrate an approach that integrates experimental data, ML models and the Maxwell model to identify promising MOF/polymer MMMs. This approach was developed to be generalizable to any possible polymer and any possible MOF without the need to rely on experimental data from either material. This choice means that these methods have the possibility of screening large collections of materials prior to their experimental synthesis. At the same time, our approach acknowledges the strong limitations that exist in molecular simulation data for molecular diffusion in MOFs and only makes predictions about situations where simulations based on rigid MOF structures are predicted to be physically accurate. Our approach combines separate ML models for adsorption in MOFs,^63^ diffusion in MOFs,^71^ and gas permeability for polymers.^48^ To illustrate the application of this approach we studied three gas separations: CO_2_/CH_4_, O_2_/N_2_ and H_2_/He. A total of 131,722 MOF/polymer MMMs were studied, with many MMMs showing the potential to exceed the upper bounds of pure polymers. Additionally, promising MOFs were identified which worked for multiple polymers and different separations.

## 2 Methods

### 2.1 Permeability and Selectivity Calculations

We used the Maxwell model to estimate the permeability of polymer/MOF MMMs^72^
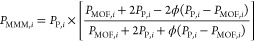
1where ϕ is the volume fraction of MOF particles, and *P*_MMM,*i*_, *P*_P,*i*_ and *P*_MOF,*i*_ are the permeabilities of the MMM, polymer, and MOF for molecule *i* respectively. Except where noted below, we used ϕ = 0.15 for all MOF/polymer MMMs. As discussed above, the Maxwell model does not account for size or shape effects associated with the filler particles and it assumes that the interfaces between polymer and MOF particles do not change the local permeability of either phase. MOF permeabilities were calculated with the solution–diffusion model^53^
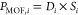
2
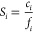
3where *D*_*i*_ is the self-diffusion coefficient, *S*_*i*_ is the solubility, *c*_*i*_ is the adsorbed gas loading, and *f*_*i*_ is the feed side partial pressure of molecule *i*. In all calculations in this paper, we set *f*_*i*_ = 1 bar. With the MMM permeability calculated, the ideal selectivity α of molecule *i* over molecule *j* is defined as
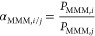
4

### 2.2 Materials Studied

All the MOFs studied in this work were taken from CoRE MOF 2019 database.^20^ 9452 MOFs were considered after removing disordered structures and structures not suitable for descriptor calculations.^71^ Specifically, AP-RDF descriptors for certain MOFs could not be calculated because the reference data lacked atomic properties for some elements. MMM properties were only predicted when the influence of MOF flexibility on molecular diffusion was predicted by the classification model of Yang and Sholl to be negligible.^71^ From the 9452 CoRE MOFs, this approach reduced the number of materials to 6527 for O_2_, 1329 for H_2_, 6912 for N_2_, 5925 for He, 6403 for CO_2_ and 4560 for CH_4_. Models that did not consider the impact of MOF flexibility on diffusion would rely on data that is predicted by the model of Yang and Sholl to be highly inaccurate for more than 44% of these CoRE MOF/gas pairs.

Experimental permeabilities and selectivities of polymers were taken from Robeson’s upper bound data.^4,73^ We studied 14 polymers for CO_2_/CH_4_, 10 for O_2_/N_2_ and 9 for He/H_2_. Detailed experimental values for the different separations can be found in the Supporting Information.

### 2.3 Simulation Methods

Molecular dynamics (MD) simulations were performed in LAMMPS.^74^ Self-diffusion simulations were carried out at 300 K and 1 atm using a Nosé–Hoover thermostat and barostat.^75^ All MD simulations were carried out with an integration time step of 1 fs. Self-diffusion coefficients were calculated from mean square displacements (MSD) using the order-*n* method.^76^ For rigid framework simulations, 10 adsorbate molecules were randomly inserted in each MOF structure using the “create_atoms” command in LAMMPS. Simulations were performed in the canonical (*NVT*) ensemble, with 2 ns equilibration phase followed by at least 10 ns of production time.^77^ For flexible framework simulations, structures were first optimized iteratively using a previously reported procedure.^78^ After structural optimization, MD simulations of the empty MOFs performed in the isothermal–isobaric (*NPT*) ensemble were carried out for 1 ns for equilibration and 2 ns for production.^70^ The final framework configuration from *NPT*-MD structure relaxation was used for subsequent self-diffusion calculations. In flexible framework simulations, 10 adsorbate molecules were randomly inserted in each simulation cell. Five independent MD simulations were performed for each system, each comprising 10 ns equilibration, followed by 40 ns of production run.^70^ We used the TraPPE-united atom (UA) force field^79^ for CO_2_ and CH_4_ and UFF4MOF^80^ for all MOF framework atoms. The Lorentz–Berthelot mixing rules were used to derive cross-interaction parameters between CO_2_ and CH_4_ and MOF framework atoms. A cutoff distance of 12.5 Å with tail corrections was applied. Simulation cells were expanded such that the dimensions along each lattice vector are at least 26 Å to satisfy the minimum image convention with respect to the cutoff distance.

Loadings of adsorbates in MOFs were simulated by grand canonical Monte Carlo (GCMC) at 300 K and 1 bar using RASPA.^81^ The universal force field (UFF) was used for MOF framework atoms.^82^ CH_4_ force field parameters were taken from TraPPE-UA and CO_2_ force field parameters were taken from TraPPE-small.^79^ Adsorbate/MOF interactions were modeled with Lennard-Jones interactions with Lorentz–Berthelot mixing rules and a cutoff of 14 Å with tail corrections. Electrostatic interactions were calculated using Ewald summation with a relative precision of 10^–6^. MOF partial charges were assigned using DDEC method.^83^ Translation, rotation and reinsertion moves were used with equal probability in these GCMC simulations.

### 2.4 Machine Learning Models

Yang et al.^71^ developed three ML models for the classification of the impact of MOF flexibility on molecular diffusion. We used the best performing *K*-nearest neighbor (KNN) model in this work. Similarly, three algorithms were tested by Yang et al. for self-diffusivity coefficient predictions, and we used their resulting gradient boosting regressor (GBR) model in this paper. The ML models predicting adsorbate loadings in MOFs were based on the ML models we have reported elsewhere.^63^ Our previous work included a classification model predicting whether non-negligible levels of adsorption exist in a MOF, but for simplicity we did not incorporate this model into the calculations reported below. In the development of the loading regression model used in this paper, the Gaussian integral descriptors developed by Choi et al.^84^ were used instead of potential energy surface (PES) descriptors, but the model architecture and other descriptors were left unchanged from our previous work.^63^ The loading ML models were trained on isotherms simulated under the assumption of MOFs being rigid. Details of the loading models can be found in our previous publications. All MOF ML models were developed in Python using scikit-learn package.^85^ We used the Polymer Genome to make predictions of *P*_P,*i*_.^48^ All of the ML models described above focus on materials properties at room temperature only, so it is not possible to use the current models to examine other temperatures.

### 2.5 Workflow of ML Models and the Maxwell Model

**Figure 1:**
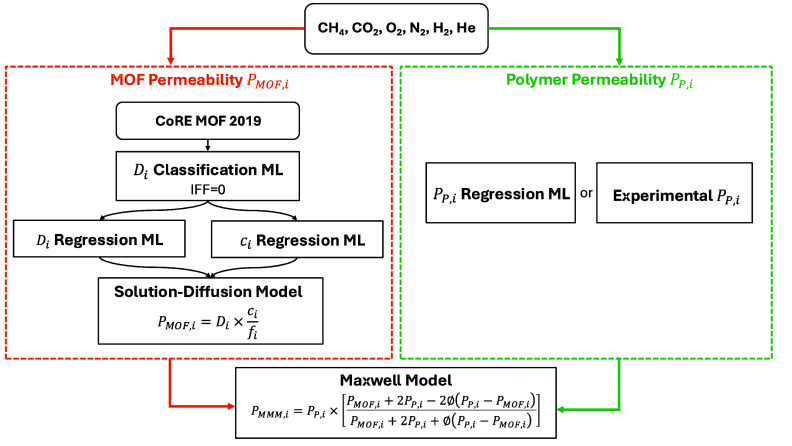
Workflow of this study.

To predict self-diffusion coefficients of adsorbates in MOFs (*D*_*i*_), we first used the classifier trained by Yang et al.^71^ to predict whether MOF flexibility has a negligible influence on adsorbate diffusion. For the MOF/adsorbate pairs where MOF flexibility has a negligible influence (IFF = 0 in the model), self-diffusion coefficients were predicted using the regression model trained in the same publication. We set up a guide to use all the models and make predictions of MMM properties (https://github.com/tdytjd/MMM_flexibility). The workflow for our predictions is shown in Figure 1.

## 3 Results

### 3.1 CO_2_/CH_4_ Separation

CO_2_/CH_4_ mixtures are commonly found in natural gas and biogas.^86,87^ Separating carbon dioxide from methane is important to increase energy density and prevent corrosion.^88^ Current methods for carbon dioxide removal often involve liquid solvents such as amine solutions, but these methods suffer from high regeneration energy and susceptibility to oxidative and thermal degradation.^89−92^ Membrane-based CO_2_/CH_4_ separations have been widely studied, and we use this separation task as an example to demonstrate our workflow.

#### 3.1.1 CO_2_/CH_4_ Using Experimental Polymer Permeabilities

In 2008, Robeson updated the CO_2_/CH_4_ permselectivity upper bound using data including two polymers of intrinsic microporosity (PIM-1 and PIM-7) to define a new upper bound.^4,93^ PIMs exhibit high permeability with moderate selectivity due to their relatively rigid but contorted molecular structures and chemical functionality. In 2019, Comesaña-Gándara et al. reported several benzotripycene-based PIM membranes that demonstrated both high permeability and selectivity.^73^ More importantly, these new PIMs suggested a new upper bound for CO_2_/CH_4_ separation, now known as the 2019 upper bound. We selected two membranes from that work, PIM-TFM-Btrip and PIM-DTFM-Btrip, to illustrate the performance space of MMMs and searching for promising MOFs for MMMs that exceed the 2019 upper bound. Permeabilities and selectivities for PIM-TFM-BTrip (*P*_P,CO_2__ = 33,700 barrer, α_CO_2_/CH_4__ = 14.8) and PIM-DTFM-BTrip (*P*_P,CO_2__ = 42,600 barrer, α_CO_2_/CH_4__ = 9.82) were taken from experimental measurements.

To comprehensively assess the performance possible with MMMs it is essential to understand the full range of permeability and selectivity that can be achieved. It is helpful to do this by considering several limiting cases. When the MOF permeability is much greater than the polymer permeability (*P*_MOF,CO_2__ ≫ *P*_P,CO_2__), the Maxwell model reduces to^94^
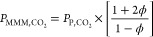
5

Conversely, if the MOF permeability is much smaller than the polymer permeability (*P*_MOF,CO_2__ ≪ *P*_P,CO_2__), the Maxwell model reduces to
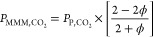
6

**Figure 2:**
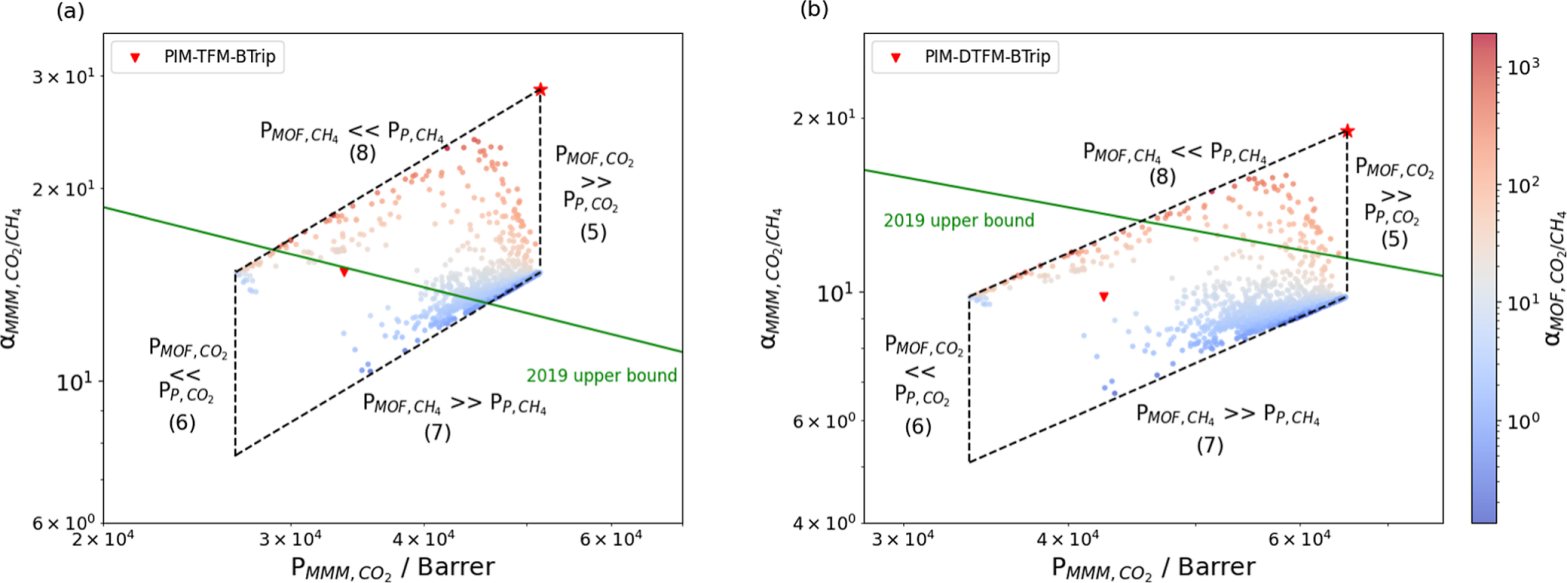
Permeability–selectivity trade-off of (a) 4278 MOF/PIM-TFM-BTrip MMMs and (b) 4278 MOF/PIM-DTFM-BTrip MMMs color coded by MOF CO_2_/CH_4_ selectivity. Both axes are in log scales. The properties of the neat polymers are indicated with red triangles, and the MMM with the maximum selectivity allowed by the Maxwell model is shown by a red star.

In both cases, the MMM permeabilities remain constant, decided solely by the polymer permeability and volume fraction. The minimum and maximum possible values of *P*_MMM,CO_2__ can be calculated using eqs 5 and 6 respectively, represented by the leftmost and rightmost vertical dashed lines in Figure 2. When *P*_MOF,CH_4__ ≫ *P*_P,CH_4__, combining eq 5 and 4 gives
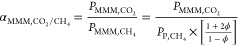
7

When *P*_MOF,CH_4__ ≪ *P*_P,CH_4__, the MMM selectivity can similarly be expressed as
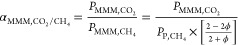
8

This set of four limiting cases defines a parallelogram on the Robeson plot as illustrated in Figure 2 for a constant filler phase fraction (ϕ = 0.15). Within the approximation of using the Maxwell model, no filler can achieve a MMM with a performance that lies outside this parallelogram. By equating eqs 5 and 8 we obtain the selectivity of the MMM that extends perpendicular to the Robeson upper bound to the greatest possible extent
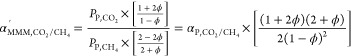
9With ϕ = 0.15, this estimate gives α_MMM,CO_2_/CH_4__^′^ = 1.93α_P,CO_2_/CH_4__, meaning that no filler can define a MMM that more than doubles the selectivity of the neat polymer.

The analysis above does not provide any indication of whether MOFs exist with the properties leading to MMMs in the preferable regions of the parallelograms in Figure 2. Our ML predictions gave 4278 MOFs with valid permeability results for both CO_2_ and CH_4_, each indicated by a data point in Figure 2. For both polymers we considered there are many MOFs that give MMMs with higher permeability but lower selectivity than the neat polymer. There are a smaller number of MOFs that increase both the permeability and selectivity in a MMM relative to the original polymer.

**Figure 3:**
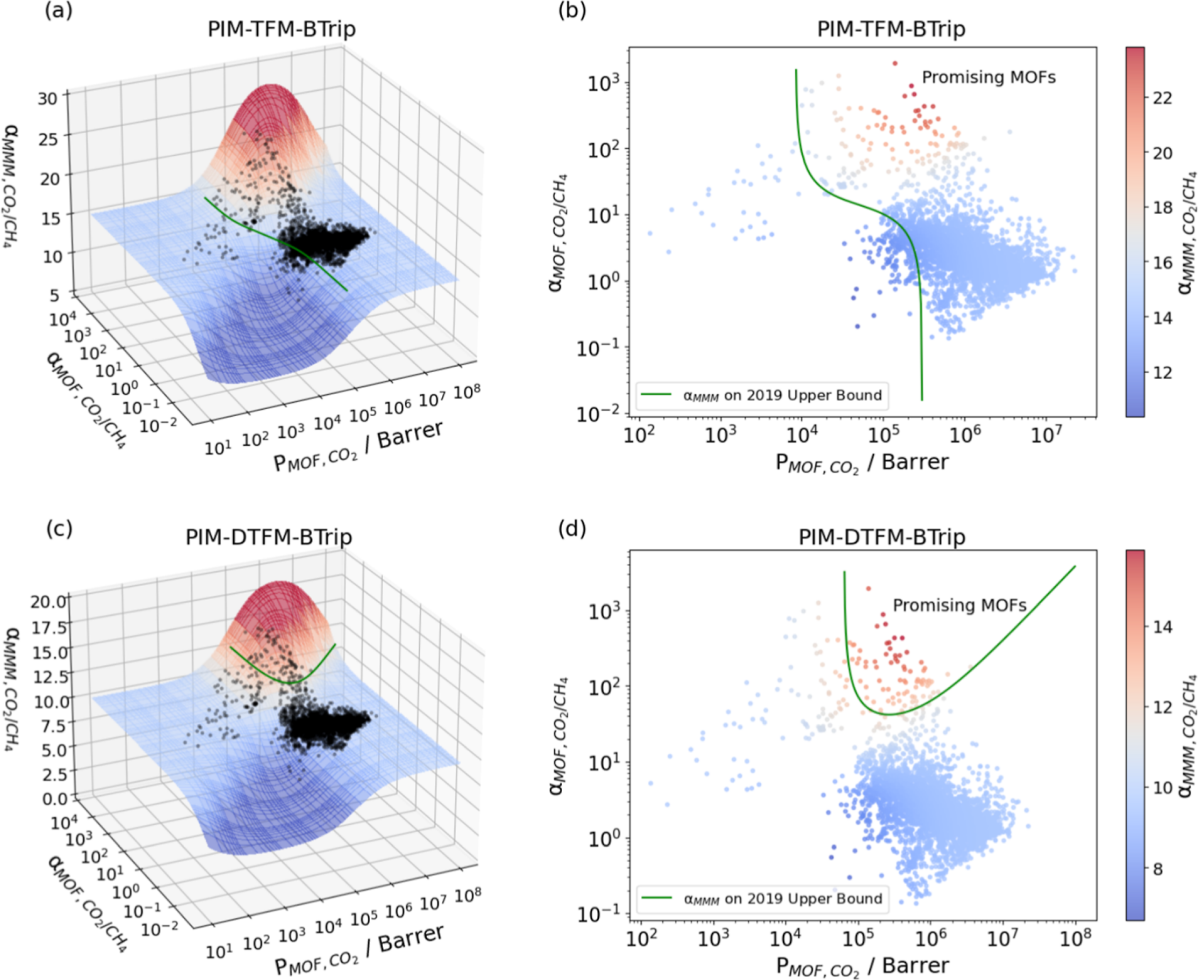
Relationships among MOF CO_2_ permeability, MOF CO_2_/CH_4_ selectivity and MMM CO_2_/CH_4_ selectivity of (a,b) 4278 MOF/PIM-TFM-BTrip MMMs and (c,d) 4278 MOF/PIM-DTFM-BTrip MMMs. The surface shows the possible MMM CO_2_/CH_4_ selectivities calculated using the Maxwell model with a fixed MOF volume fraction. MMMs using the MOFs above the green curves in (b,d) are above the 2019 upper bound.

An alternative view of this data is shown in Figure 3, showing the MMM selectivities as a function of the MOF properties. The shaded surface in Figure 3a,c shows the full space of selectivities possible within the Maxwell model, with the ML predictions for individual MOFs shown as black symbols. Figure 3b,d show the predicted MMM selectivity for all the MOFs we considered along with a green curve that defines the Robeson upper bound. The shape of the green curve is different for a polymer lying on the upper bound (Figure 3b) and a polymer lying below the upper bound (Figure 3d). For the purposes of discussion below, we label all MOFs that yield MMMs predicted to lie above the Robeson upper bound for a particular polymer as “promising” MOFs. PIM-TFM-BTrip was very close to the upper bound, allowing us to identify 4037 promising MOFs. For PIM-DTFM which lies below the upper bound, only 79 MOFs were considered promising by this definition.

**Figure 4:**
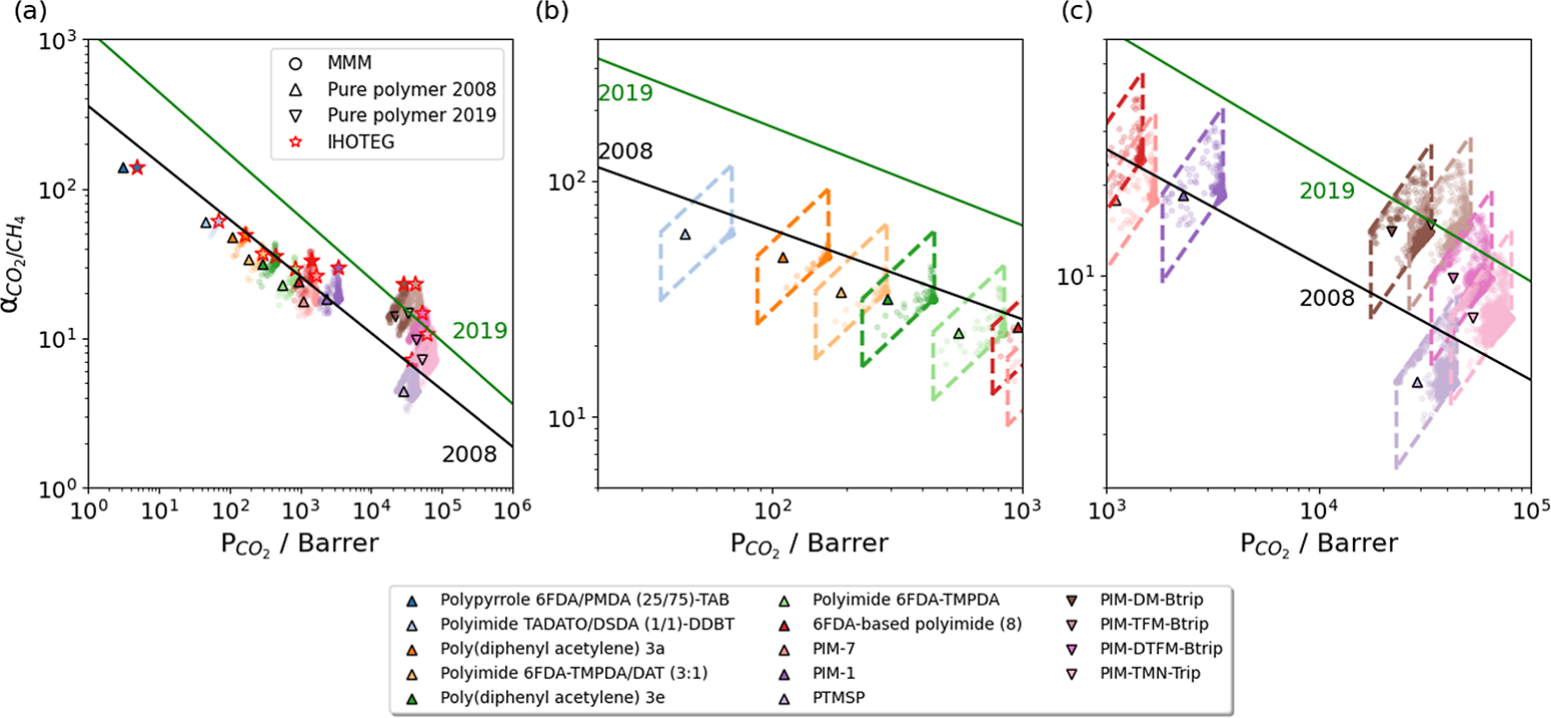
(a) Predicted MMM permeability and CO_2_/CH_4_ selectivity for 4278 MOFs combined with 14 polymers, along with an expanded view for of polymers with (b) low CO_2_ permeability and (c) high CO_2_ permeability. Solid lines indicate the 2008 and 2019 upper bounds. MMMs associated with a specific MOF, structure code IHOTEG, are shown in (a) with red stars. Dashed lines indicate bounds on the possible performance of MMMs within the Maxwell model at the chosen filler fraction.

We performed similar calculations for 12 more polymers known from experiments, including 10 polymers close to the 2008 upper bound and 2 polymers close to the 2019 upper bound. The permeabilities and selectivities of these polymers are provided in the Supporting Information. The resulting predictions for ∼60,000 distinct MMM compositions are shown in Figure 4. For polymers with low CO_2_ permeabilities, nearly all MOF permeabilities were much larger than the polymer permeabilities, placing the MMMs in the bottom-right corner of the parallelograms defined by the limiting cases of the Maxwell model. For polymers with high CO_2_ permeabilities, gas permeabilities of MOFs and polymers were more closely matched, so more MOF/polymer MMMs exceeded the upper bound and spread toward the ideal MOF/polymer MMM (i.e., the top right vertex of the parallelogram, Figure 4c). Notably, exceeding the 2019 upper bound remained challenging when using polymers near the 2008 upper bound.

Table 1 lists six MOFs for which the MOF/polymer MMM was predicted to exceed the upper bound for more than half of the polymers. They all exhibited high CO_2_ permeability and high CO_2_/CH_4_ selectivity. As an example, IHOTEG is illustrated in Figure 4a. The observed high selectivity from the ML predictions can be primarily attributed to the significant differences in solubilities between CO_2_ and CH_4_.

**Table 1:** MOF Permeabilities and Selectivities Calculated from Simulated and ML Predicted Properties of the Six Promising MOFs for CO_2_/CH_4_ MMM

MOF name	LCD (Å)	PLD (Å)	*P*_MOF,ML,CO_2__ (barrer)	α_MOF,ML,CO_2_/CH_4__
CIQYUX	3.59	2.61	1.12 × 10^4^	7.73 × 10^2^
FOHRUR	3.40	2.45	1.77 × 10^4^	9.69 × 10^2^
IHOTEG	3.67	2.49	1.40 × 10^5^	1.92 × 10^3^
SIZNIA	3.62	2.53	2.22 × 10^5^	8.76 × 10^2^
XALVOY	3.43	2.89	1.33 × 10^4^	7.31 × 10^2^
XULRIH	3.53	2.52	2.85 × 10^4^	1.24 × 10^3^

#### 3.1.2 MMMs for CO_2_/CH_4_ Separations Using ML Polymer Permeabilities

To demonstrate the integration of ML-based polymer properties into our workflow, we used the Polymer Genome^95^ to make predictions for a subset of the data set of MMM experimental data from Guan et al.^23^ Our analysis focused on the 98 MMM CO_2_/CH_4_ experiments in this data set that had with complete pressure and temperature documentation, homopolymers as the polymeric phase, unfunctionalized MOFs, MOF volume fraction ≤0.3, and a negligible predicted influence on diffusion from MOF flexibility (as predicted by the diffusion ML classifier described above). The ML models we used for MOFs were developed on data simulated at 300 K, while the experimental temperatures varied from 293 to 308 K. The data were collected from different research groups, and the experimental conditions among the various studies were not identical. Besides the temperature, the thickness of the membranes, feed pressures and MOF loadings were all slightly different. When making predictions, the MOF loadings and pressures associated with each experimental measurement were used. More information on this data set is given in the Supporting Information. The experimental data included a wide range of MMMs, with many of them close or above the upper bound (Figure S1).

**Figure 5:**
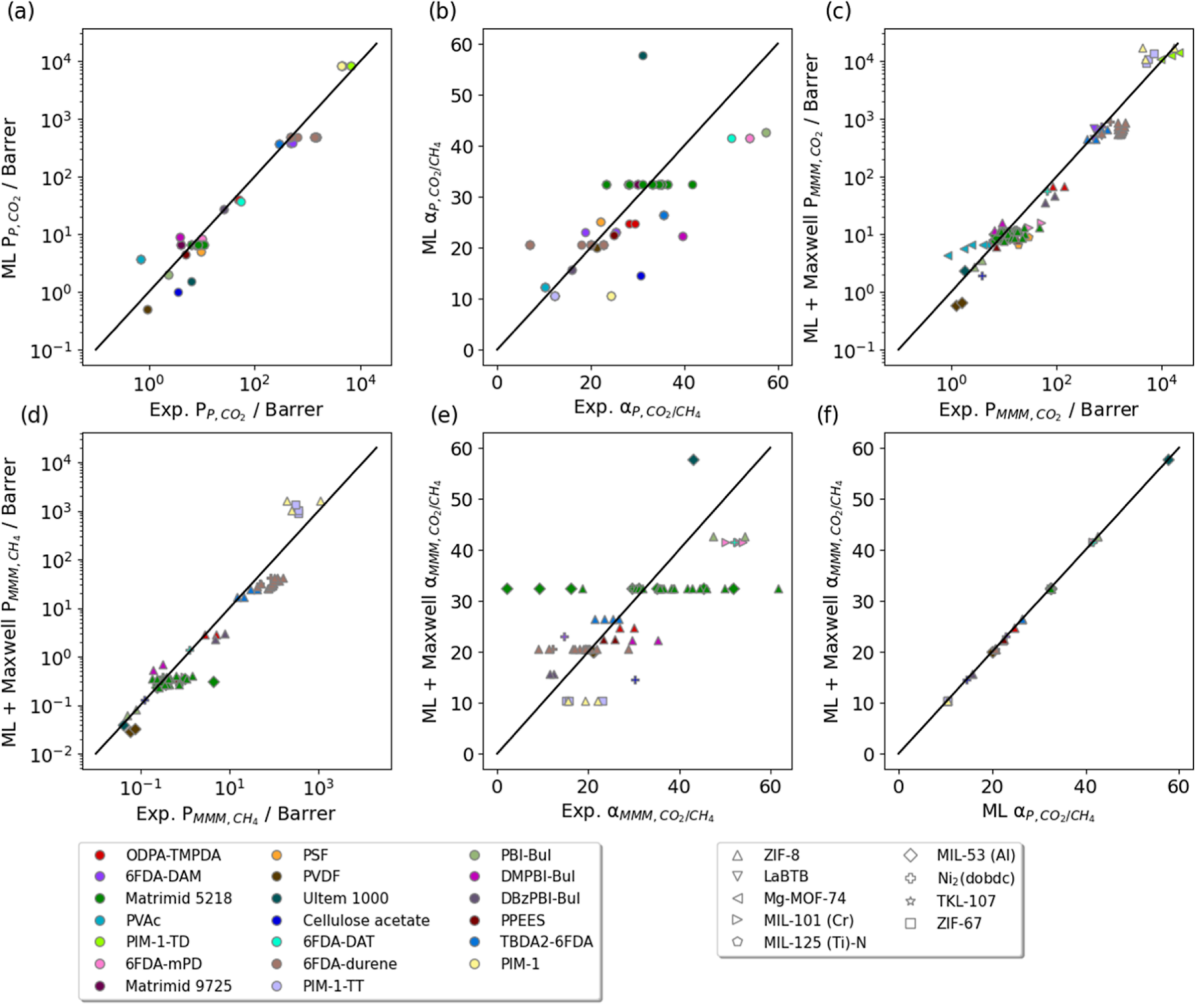
(a) Parity plot of polymer CO_2_ permeabilities from experiments and ML predictions. (b) Parity plot of polymer CO_2_/CH_4_ selectivities from experiments and ML predictions. (c) Parity plot of MMM CO_2_ permeabilities from experiments and from ML predictions and the Maxwell model. (d) Parity plot of MMM CH_4_ permeabilities from experiments and from ML predictions and the Maxwell model. (e) Parity plot of MMM CO_2_/CH_4_ selectivities from experiments and from ML predictions and the Maxwell model. (f) Parity plot of ML predicted polymer CO_2_/CH_4_ selectivities and MMM CO_2_/CH_4_ selectivities calculated from ML predictions and the Maxwell model.

The agreement between the Polymer Genome predictions and experimental data for the 20 distinct polymers in the data set is shown in Figure 5a, giving *r*^2^ = 0.94 for log(*P*_P,CO_2__) and 0.99 for log(*P*_P,CH_4__). We note, however, that the ML predictions for selectivities in the neat polymers are less accurate (Figure 5b, *r*^2^ = 0.58). For the 98 MMMs, permeabilities predicted from our ML predictions and the Maxwell model were also accurate, with *r*^2^ = 0.94 for log(*P*_P,CO_2__) (Figure 5c) and *r*^2^ = 0.92 for log(*P*_P,CH_4__) (Figure 5d).

Similar to the situation with pure polymers (Figure 5b), the predicted MMM selectivities show more variation relative to the experimental data (*r*^2^ = 0.46, Figure 5e). For MMM selectivities, the predictions for different MMMs using different MOFs and same polymers were nearly the same, consistent with the nature of the Maxwell model. In this data set, the CO_2_ and CH_4_ permeabilities of all the polymers considered were orders of magnitude smaller than the predicted permeabilities of the MOFs (Figure S2a,b). Within the Maxwell model, when *P*_MOF_ ≫ *P*_P_, the selectivities of any MMM and the original polymers are the same, lying in the lower-right vertices of the parallelograms in Figures 2 and 3
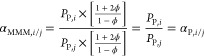
10

This approximation held for all MMMs in this data set (Figure 5f), giving rise to the horizontal groupings of data for each polymer in Figure 5e.

The results in Figure 5e point to underlying uncertainties in the experimental data. For example, the CO_2_/CH_4_ experimental selectivities of pure Matrimid 5218 varied from 20 and 40 (Figure 5b), already a considerable range. If the Maxwell model is approximately correct, the analysis above shows that MMMs made from this polymer should have essentially the same selectivities for every MOFs used in this data set. This is clearly not the case for the experimental data in Figure 5e, which includes MMM selectivities varying from 6.7 to 47.5. Even MMMs with the same MOF/polymer combination [for example MIL-53 (Al)/Matrimid 5218], the selectivities ranged widely, with values from 6.7 to 17.0. Teesdale et al. showed that the sample-to-sample variation was substantial enough to mask any differences in permeability and selectivity between two types of 6FDA-Durene MMMs with low and high porosity UiO-66-NH_2_, highlighting the importance of conducting and reporting replicate experiments for composite membranes.^96^ Individually, the data points in Figure 5e can be interpreted as pointing to deviations from the assumptions of the Maxwell model. Collectively, the variation in this experimental data points to ongoing challenges in understanding and controlling the factors that lead to sample-to-sample agreement in experiments with MOF-based MMMs.

The discussion of the Maxwell model above indicates that increasing the selectivity of a MMM relative to a neat polymer is best achieved when the MOF permeabilities and polymer permeabilities have similar orders of magnitude. However, there was insufficient experimental data on MMMs using polymers with very high permeabilities to confirm if the selectivities of these MMMs differ from polymer selectivities. It is therefore not currently possible to unambiguously determine whether our workflow would accurately predict MMM properties in these cases by direct comparison to experimental data.

### 3.2 O_2_/N_2_ Separation

**Figure 6:**
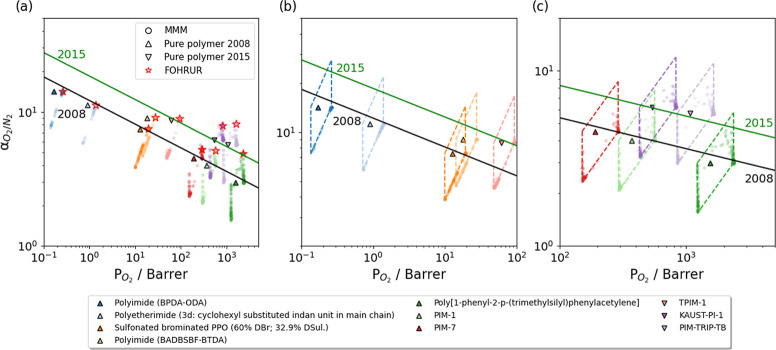
(a) Predicted MMM permeability and O_2_/N_2_ selectivity for 6477 MOFs combined with 10 polymers, along with an expanded view for of polymers with (b) low O_2_ permeability and (c) high O_2_ permeability. Solid lines indicate the 2008 and 2015 upper bounds. MMMs associated with a specific MOF, structure code FOHRUR, are shown in (a) with red stars.

Oxygen and nitrogen are among the most significant industrial gases. Their high demand makes the O_2_/N_2_ separation the most studied gas pair.^4,97^ We applied our workflow to 10 polymers and 6477 MOFs to screen materials for this separation (Figure 6). Seven of the 10 polymers were taken from the 2008 upper bound. Similar to the polymers developed for CO_2_/CH_4_ separation, several PIMs with good performance were synthesized leading to a revised 2015 upper bound.^98^ Three remaining polymers were taken from the 2015 upper bound. Details of the polymers can be found in the Supporting Information.

When polymer O_2_ permeabilities were relatively low, all MMMs were scattered on the bottom lines of the parallelograms (Figure 6b). There were no MOFs with predicted *P*_MOF,N_2__ < 50 barrer, which meant that *P*_MOF,N_2__ ≫ *P*_P,N_2__ (eq 7) for all cases we considered. There would be essentially no improvement in selectivity relative to the neat polym for all these MOF/polymer MMMs. When polymer O_2_ permeabilities were higher (Figure 6c), most MMMs were scattered on the two vertical edges (left: *P*_MOF,O_2__ ≪ *P*_P,O_2__, right: *P*_MOF,O_2__ ≫ *P*_P,O_2__). 67 MOFs had predicted *P*_MOF,O_2__ < 10^2^ barrer, and MMMs with these MOFs and polymers with high O_2_ permeabilities would not be favorable. Fortunately, most of the MOFs showed high permeabilities and would improve the permeability of the MMMs. This observation again emphasized the necessity to match the permeabilities of the MOFs and the polymers. We identified 54 MOFs for which the MOF/polymer MMM exceeded the upper bound for more than seven of the ten polymers. FOHRUR, which was proposed above as promising in CO_2_/CH_4_ separations, was again identified for O_2_/N_2_ separation. Our model predicts that a FOHRUR/poly[1-phenyl-2-*p*-(trimethylsilyl)phenylacetylene] MMM exceeds the 2015 upper bound while using a polymer near the 2008 upper bound. However, other MMMs using polymers near the 2008 upper bound did not exceed the new upper bound.

### 3.3 He/H_2_ Separation

Helium is essential due to its chemical inertness, ultralow boiling point and light mass. However, the extraction of He from natural gas using cryogenic distillation is energy intensive.^99^ Separating He from hydrogen, which is another common natural gas component, is particularly challenging due to their similar sizes and condensation properties. Perfluorinated polymers showed good performance for He/H_2_ separation, but the reported selectivities of these polymers were all lower than 5.^4^ We selected 9 polymers near the 2008 upper bounds and did the same analysis as above for this separation using our workflow.

**Figure 7:**
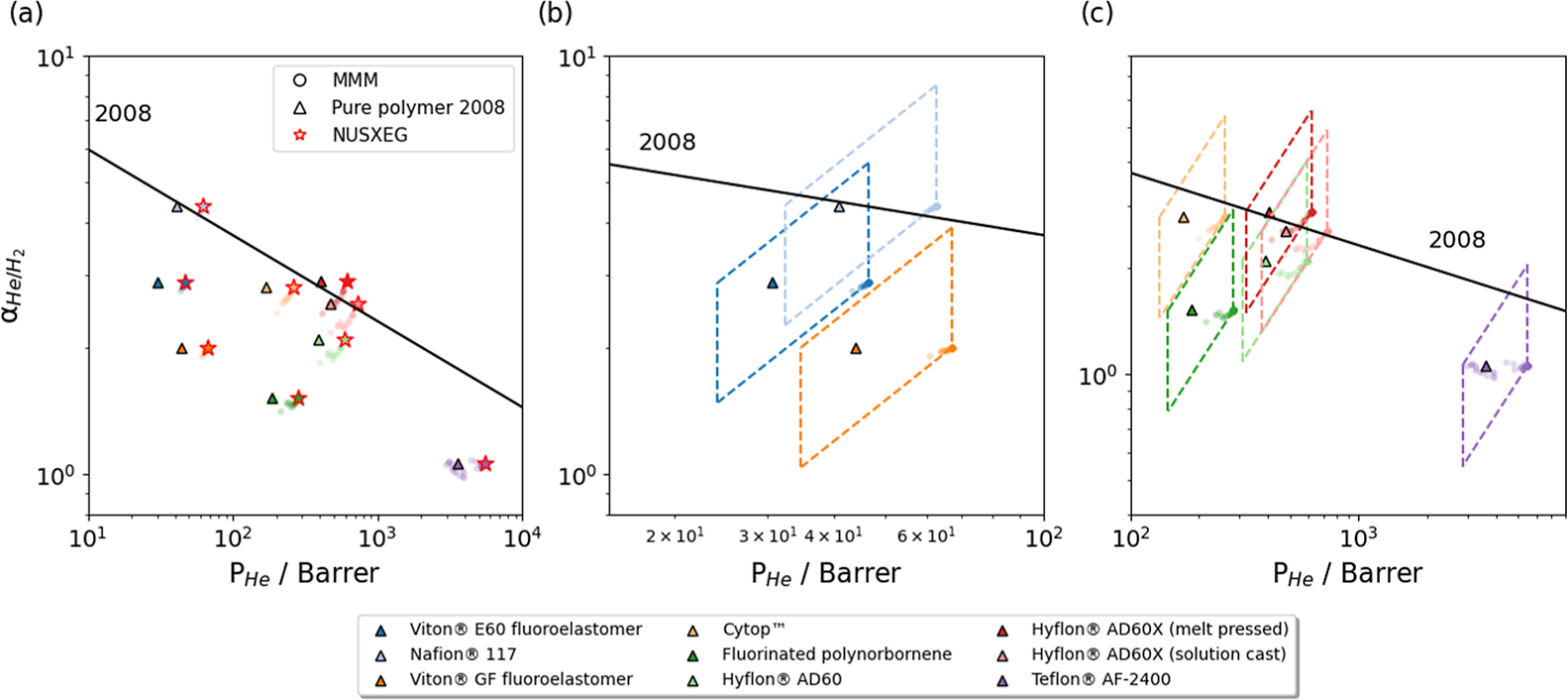
(a) Predicted MMM permeability and He/H_2_ selectivity for 790 MOFs combined with 9 polymers, along with an expanded view for of polymers with (b) low He permeability and (c) high He permeability. Solid line indicates the 2008 upper bound. MMMs associated with a specific MOF, structure code NUSXEG, are shown in (a) with red stars.

MOF permeabilities of both He and H_2_ were predicted to be significantly larger than polymer permeabilities. Most MMMs in Figure 7 were clustered at the lower right vertices of the parallelograms defined by the Maxwell model. There is a nearly horizontal line of MOF/Teflon AF-2400 MMMs in Figure 7c. When α_P,He/H_2__ = α_MOF,He/H_2__, the Maxwell model predicts that α_MMM,He/H_2__ = α_P,He/H_2__ = α_MOF,He/H_2__. Most α_MMM,He/H_2__ were similar to the pure Teflon AF-2400 selectivity (1.06), since 733 out of 790 MOFs had 0.7 < α_MOF,He/H_2__ < 1.3.

We observed no significant improvement in selectivity for this gas pair for nearly all MMMs, as most MOFs exhibited both high and similar permeabilities for He and H_2_. Consequently, we were unable to identify any promising MOFs (using the definition above that such a MOF would yield a MMM lying above the upper bound for more than half of the polymers). These findings suggested that MOF-based MMMs might not be an ideal choice for this separation task if improving selectivity is the primary objective.

### 3.4 Molecular Simulations of Six Promising MOFs for CO_2_/CH_4_ Separations

The results above are examples of using ML models to explore large collections of materials. Although this approach can be useful for initial screening of materials, it is typically difficult to place firm bounds on the precision of these models. It is therefore advisible to couple the predictions of these models with more detailed methods. As an example of this strategy, we used molecular simulations to examine the viability of the materials listed above in Table 1 for CO_2_/CH_4_ separations. More specifically, we performed molecular simulations of CO_2_ and CH_4_ adsorption and diffusion in each of the MOFs listed in Table 1.

The largest cavity diameters (LCD) and pore limiting diameters (PLD) of the six MOFs listed in Table 1 are relatively small compared to the kinetic diameter of CO_2_ (3.3 Å), and the PLDs of all six MOFs are smaller than the kinetic diameter of CH_4_ (3.8 Å). The predictions above relied on ML models for diffusivities in MOFs that did not necessarily include extensive training data with these characteristics; among the MOFs used for training these models^71^ only four MOFs had PLD <3.3 Å, and 4% of the MOFs had PLD <3.8 Å (Figure S3). To investigate in more detail whether CO_2_ and CH_4_ can effectively diffuse through these MOFs, MD simulations were used to calculate self-diffusivities at low loadings in both rigid and flexible frameworks. In each system, we investigated whether the adsorbed molecules diffuse within the time scales accessible in our MD simulations. Self-diffusivities are only reported for systems in which the MSD of the adsorbed molecules exceeds a threshold value of 14^2^ Å^2^ within a simulation time of 10 ns.^100^ If the MSD remained below this threshold, we describe the adsorbed molecules as not diffusing, although it would be more precise to say that the diffusivity was less than ∼10^–7^ cm^2^ s^–1^. An example is shown in Figure S4. We emphasize that throughout our work, diffusion coefficients from molecular simulations with fully flexible MOFs are considered the “ground truth”, so the accuracy of more approximate methods including simulations with rigid MOFs or ML predictions should wherever possible be compared to these values.

The results of our diffusivity simulations are summarized in Table 2. A striking feature of these simulations is that measurable diffusion was only observed in a few of the examples, contrary to the ML diffusivity predictions that every example would have a diffusivity readily observable in our simulations. It would be useful to understand in a direct way how this disagreement between the ML predictions and simulation data came about. Unfortunately, the training and test data for the diffusion coefficient regressor ranged from 10^–7^ to 10^–2^ cm^2^/s,^71^ indicating that the ML model was only validated for cases where diffusion was observed in molecular simulations. It is not straightforward to assess whether this prediction is accurate for the six MOFs we examined because for most of the examples our molecular simulations did not give reliable values for both diffusivities. A more effective approach would be to first train a classifier to identify cases where diffusion occurs in molecular simulations and then apply the regressor solely to those case for more accurate predictions.

**Table 2:** Simulated and ML Predicted Self-Diffusivity Comparisons of the Six Promising MOFs for CO_2_/CH_4_ MMM, Showing Results Predicted from ML (“ML”), and Observed with Molecular Simulations of Rigid MOFs (“Rigid”) and Flexible MOFs (“Flexible”)^a^

MOF name	*D*_CO_2_,ML_ (×10^–5^ cm^2^/s)	*D*_CO_2_,rigid-MD_ (×10^–5^ cm^2^/s)	*D*_CO_2_,flexible-MD_ (×10^–5^ cm^2^/s)	*D*_CH_4_,ML_ (×10^–5^ cm^2^/s)	*D*_CH_4_,rigid-MD_ (×10^–5^ cm^2^/s)	*D*_CH_4_,flexible-MD_ (×10^–5^ cm^2^/s)
CIQYUX	4.79	x	x	1.09	x	x
FOHRUR	10.3	1.12	x	2.61	x	x
IHOTEG	9.58	x	x	2.06	x	x
SIZNIA	13.5	x	0.41	3.01	x	x
XALVOY	12.3	x	x	4.41	x	x
XULRIH	17.0	x	7.87	3.66	x	4.10

a x: no diffusion observed in MD simulations.

We also compared the simulated and ML predicted CO_2_ and CH_4_ loadings in the same six MOFs (see Table 3). Since CH_4_ was included in the training set of the MOF loading ML models,^63^ the loading predictions for CH_4_ agreed well with the GCMC simulation results. Although the GCMC simulations for CO_2_ loadings were less consistent with the ML predictions, the simulated CO_2_ loadings were still significantly higher compared to CH_4_.

**Table 3:** Simulated and ML Predicted Loading Comparisons at 1 bar of the Six Promising MOFs for CO_2_/CH_4_ MMMs

MOF name	*c*_CO_2_,ML_ (mol/kg)	*c*_CO_2_,GCMC_ (mol/kg)	*c*_CH_4_,ML_ (mol/kg)	*c*_CH_4_,GCMC_ (mol/kg)
CIQYUX	4.73 × 10^–2^	4.46 × 10^–3^	2.70 × 10^–4^	2.50 × 10^–4^
FOHRUR	4.15 × 10^–2^	1.29 × 10^–1^	1.70 × 10^–4^	6.60 × 10^–4^
IHOTEG	3.80 × 10^–1^	7.34 × 10^–2^	9.20 × 10^–4^	1.79 × 10^–3^
SIZNIA	4.31 × 10^–1^	1.77 × 10^0^	2.21 × 10^–3^	4.32 × 10^–3^
XALVOY	2.19 × 10^–2^	1.68 × 10^0^	8.00 × 10^–5^	8.00 × 10^–5^
XULRIH	3.06 × 10^–2^	2.12 × 10^–1^	1.10 × 10^–4^	1.00 × 10^–4^

An interesting outcome from the data above is that based directly on molecular simulations we are only able to make numerical predictions for the properties of MMMs for only one of the six MOFs, namely the MOF with structure code XULRIH. For this MOF, diffusivities of both CO_2_ and CH_4_ are available from our MD simulations with flexible structures. If we consider the polymer with the lowest CO_2_ permeability we examined above, polypyrrole 6FDA/PMDA (25/75)-TAB, our ML approach predicted a CO_2_ permeability of 4.79 barrer and CO_2_/CH_4_ selectivity of 140.2 for the XULRIH/polypyrrole 6FDA/PMDA (25/75)-TAB MMM. Using molecular simulations to give the adsorption and diffusion properties of this MOF, the predicted MMM permeability and selectivity are 4.79 barrer and 140.0. Similarly, for the polymer with the highest CO_2_ permeability, PIM-TMN-Trip, our ML model predicted XULRIH/PIM-TMN-Trip MMM CO_2_ permeability of 4.86 × 10^4^ barrer and CO_2_/CH_4_ selectivity of 8.47, while molecular simulation data gives values of 5.76 × 10^4^ and 9.93.

Our results illustrate positive and negative aspects of using ML models to screen MOFs as components in MMMs. Of the six MOFs we examined in detail, precise data from molecular simulations was only accessible for one MOF, implying that the accuracy of the ML predictions for the remaining five materials is unclear. For the remaining MOF, XULRIH, detailed molecular simulations indicate that this material is indeed an interesting candidate material for CO_2_/CH_4_ separations with MMMs. We must point out, however, that the ML model we used to predict molecular diffusivities in rigid MOFs made inaccurate predictions for XULRIH but that these predictions were in reasonable agreement with the more physically meaningful simulations with a flexible MOF. In other words, our screening approach did lead to identification of an interesting material that may not otherwise have been studied, but not because the underlying ML models made highly accurate predictions for this material.

During the curation of the CoRE MOF 2019 database, MOFs structures were automatically cleaned.^20^ However, this process can sometimes generate problematic structures.^101^ We carefully compared the stoichiometry of structures from the CoRE MOF 2019 database with their experimentally reported structures (Table S4). For the first five MOFs in Table 3, the CoRE MOF 2019 structures were reasonable, since only water molecules were removed during the cleaning process. However, we identified missing H atoms in XULRIH, highlighting the need for caution when working with materials generated by high-throughput processes.

## 4 Discussion and Conclusions

We demonstrated a workflow that integrated machine learning models with the consideration of MOF flexibility enabling prediction of permeability and selectivity of numerous MOF/polymer MMMs for different separation tasks. By combining the Maxwell model and ML models that can be applied to any material composition, this approach provides a useful method for screening materials in gas separation applications. The use of this approach was illustrated with results from 131,722 MMMs for several gas separations. In addition to predictions for specific polymer/MOF combinations, we showed how analysis of the Maxwell model can define an envelope within which the performance of an MMM with any filler must lie. This analysis helps clarify the observation that it is important for the permeability of a polymer and a filler to be reasonably well matched for a MMM to be effective.

Despite its generality, our workflow has certain limitations. First, the Maxwell model assumed ideal interfaces between MOFs and polymers and does not consider the effects of MOF size, shape and packing. Although alternative models may offer better accuracies for perm-selectivity predictions,^35^ they require additional system-specific parameters and significantly more effort. The interfacial engineering of MOF-based MMM has been investigated by molecular dynamics.^102^ However, this process requires careful design and parametrization of the interface and is beyond the scope this work.

A key limitation of our approach is that it uses ML models for solubilities and diffusivities in MOFs, not more detailed data from molecular simulations. Although these prior ML models were developed with care, there is no guarantee that their training data is strongly represented with materials and molecules in the specific regimes that are of greatest interest for MMMs. Fortunately, it is possible to directly assess the predictions of the ML models for the most interesting materials by identifying a small number of materials of special interest and then performing detailed molecular simulations for those materials.^103^ We illustrated this approach for CO_2_/CH_4_ separations by performing molecular simulations for six small pore MOFs that were predicted by our ML-based approach to be useful in MMMs with a range of polymers. Of these six MOFs, only one exhibited molecular diffusivities fast enough to be readily observed for both molecules in molecular simulations. This specific MOF does indeed appear to be an interesting candidate for MMM-based CO_2_/CH_4_ separations. The ambiguities in simulating diffusion in the other five MOFs suggest that the ML models we used for diffusion in MOFs may lack accuracy for small pore MOFs. At the same time, our results illustrate the challenge of generating molecular simulation data that could be used to train more accurate models.

We only focused on MOF/polymer MMMs when MOF flexibility was predicted to have a negligible influence on diffusion. It is of course possible that MOF flexibility has a strong influence on diffusion and a material is promising for separations using MMMs. Furthermore, the molecular simulations for six MOFs that we reported were all examples where MOF flexibility was predicted to have a small effect, but the molecular simulation data gave examples where this outcome was not correct. The development of more robust data sets from molecular simulations of diffusion with flexible MOFs remains a challenging and interesting problem for the future.

In this work all ML predictions and molecular simulations of adsorption loadings assumed that MOFs were rigid frameworks, but flexibility can also affect the accuracy of predicted loadings. Yu et al. used adsorption–relaxation simulations to test the assumption of rigid frameworks in CO_2_ uptake for four well-known MOFs and for seven molecules in 15 random MOFs from the CoRE MOF database.^104^ The results indicated that adsorption–relaxation simulations are essential for making accurate quantitative predictions of adsorption in many MOFs, especially at low loadings. However, calculations using rigid structures can still provide useful data. Given that framework flexibility plays a crucial role in some situations but is less important in other cases, it would be beneficial to determine beforehand when this effect can be ignored, allowing for the use of rigid framework simulations with confidence.

These findings highlight the importance of supplementing ML predictions with detailed simulations and careful experimental validation to ensure robust material selection and performance assessment. This workflow laid the groundwork for future advancements, such as gathering more reliable experimental data under desired conditions and developing more accurate ML models incorporating more practical factors. These potential improvements would broaden the workflow’s applicability to real-world challenges in material discovery.

## Supporting Information Available

ML models, PLD distributions, MSD analysis of diffusion, permeability distributions, experimental polymer permeabilities and selectivities (PDF)
Tabulated data from the figures (XLSX)
