# Supplementary material for: Facilitating Screening of MOFs for Mixed Matrix Membranes Using Machine Learning and the Maxwell Model
Source: J Phys Chem C Nanomater Interfaces. 2025 May 1;129(19):9217–30. doi: 10.1021/acs.jpcc.5c01483 (PMC12086843; doi:10.1021/acs.jpcc.5c01483)
Supplement: Supplementary file 1 — jp5c01483_si_001.pdf [file jp5c01483_si_001.pdf]

Supporting Information

Facilitating Screening of MOFs for Mixed Matrix Membranes Using Machine Learning and the Maxwell Model

Xiaohan Yu[1], Jia Yuan Chng[1] and David S. Sholl[2,*]

[1]School of Chemical & Biomolecular Engineering, Georgia Institute of Technology, Atlanta, Georgia 30332-0100, United States

[2]Oak Ridge National Laboratory, Oak Ridge, TN 37830, United States

*Corresponding author. Email: shollds@ornl.gov

Table of Contents

S1 Supplementary Figures ***S1***

S2 Supplementary Tables ***S3***

In addition to the Supporting Information below, code that implements all models described in the manuscript is available via GitHub at https://github.com/tdytjd/MMM_flexibility. The tabulated data from the figures in the main manuscript are available in Data S1.

S1 Supplementary Figures

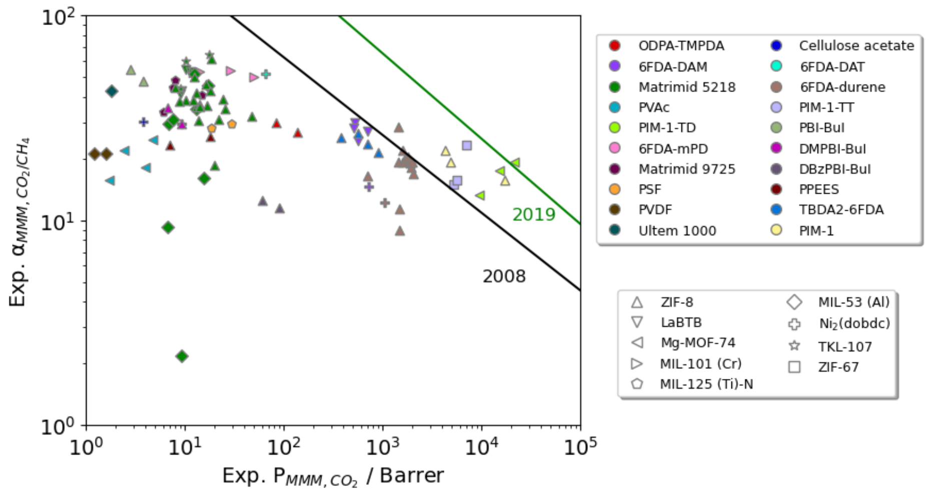

Figure S1 CO_2 permeabilities and CO_2/CH_4 selectivities of 98 MMMs

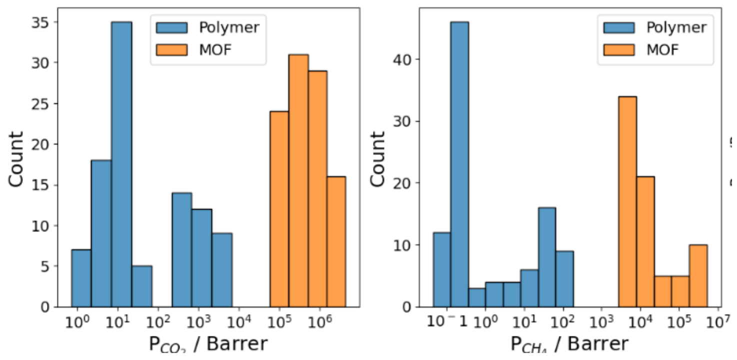

Figure S2 (a) CO_2 and (b) CH_4 MOF and polymer permeability distributions

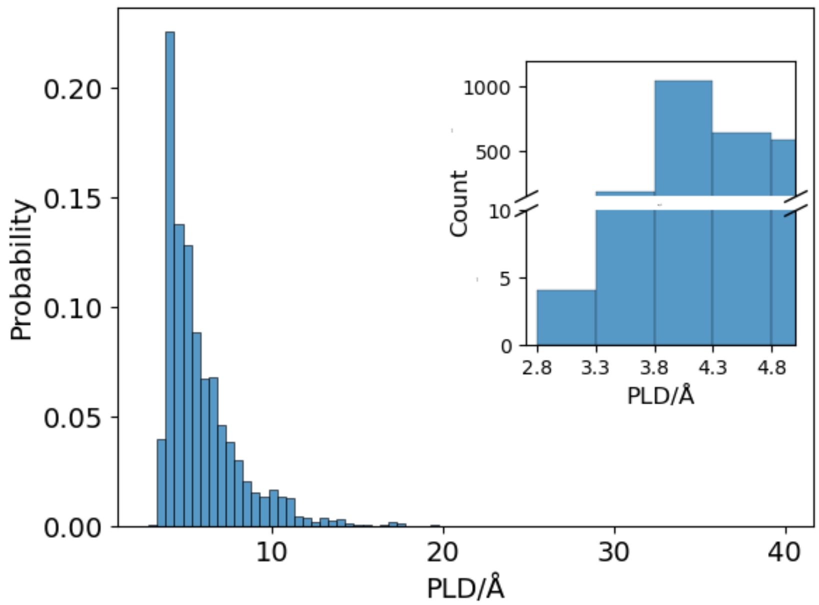

Figure S3 PLD distribution of MOFs used for diffusion ML training

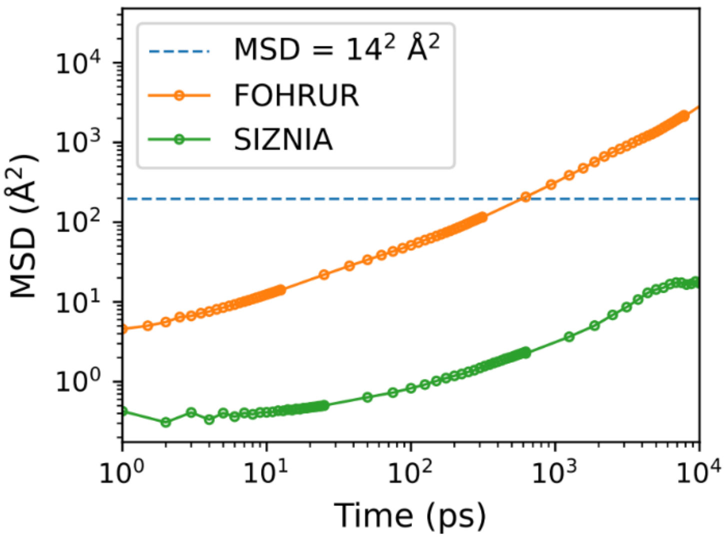

Figure S4 MSD analysis of CO_2 diffusion in rigid FOHRUR and rigid SIZNIA. The dashed horizontal line represents the threshold MSD value distinguishing diffusing and non-diffusing CO_2 within the MOFs. The threshold value corresponds to displacements of approximately half the length of the simulation cell. If the MSD of the adsorbed molecule exceeds the threshold value within a simulation time of 10 ns, we consider the adsorbed molecule as capable of diffusion in the MOF. In this figure, CO_2 readily diffuses in rigid FOHRUR but does not diffuse in rigid SIZNIA.

S2 Supplementary Tables

Table S1 Experimental polymer permeabilities and selectivities for CO_2/CH_4

Polymer	P_{P,CO_2}/Barrer	$\alpha_{P,CO_2/CH_4}$	Upper Bound	Ref.
Polypyrrole 6FDA/PMDA (25/75)-TAB	3.13	140	2008	1
Polyimide TADATO/DSDA (1/1)-DDBT	45	60		2
Poly(diphenyl acetylene) 3a	110	47.8		3
Polyimide 6FDA-TMPDA/DAT (3:1)	187.6	33.9		4
Poly(diphenyl acetylene) 3e	290	31.5		3
Polyimide 6FDA-TMPDA	555.7	22.7		4
6FDA-based polyimide (8)	958	24		5
PIM-7	1100	17.7		6
PIM-1	2300	18.4		6
PTMSP	29000	4.46		7
PIM-DM-Btrip	22000	14	2019	8
PIM-TFM-Btrip	33700	14.8		
PIM-DTFM-Btrip	42600	9.82		
PIM-TMN-Trip	52800	7.28		

Table S2 Experimental polymer permeabilities and selectivities for O_2/N_2

Polymer	P_{P,O_2}/Barrer	$\alpha_{P,O_2/N_2}$	Upper Bound	Ref.
Polyimide (BPDA-ODA)	0.17	14.2	2008	9
Polyetherimide (3d: cyclohexyl substituted indan unit in main chain)	0.9	11.2		10
Sulfonated brominated PPO (60% DBr; 32.9% DSul.)	12.6	7.4		11
Polyimide (BADBSBF-BTDA)	18	9		12
Poly[1-phenyl-2-p-(trimethylsilyl)phenylacetylene]	1550	2.98		13
PIM-1	370	4		6
PIM-7	190	4.5		6
TPIM-1	61	8.6	2015	14
KAUST-PI-1	542	6.2		
PIM-TRIP-TB	1073	5.7		

Table S3 Experimental polymer permeabilities and selectivities for He/H_2

Polymer	$P_{P,He}$/Barrer	$\alpha_{P,He/H_2}$	Upper Bound	Ref.
Viton® E60 fluoroelastomer	30.5	2.87	2008	15
Nafion® 117	40.9	4.39		16
Viton® GF fluoroelastomer	43.9	2.01		15
Cytop™	170	2.8		17
Fluorinated polynorbornene	185	1.52		18
Hyflon® AD60	390	2.1		17

Hyflon® AD60X (melt pressed)	405	2.89	[19, 20]
Hyflon® AD60X (solution cast)	476	2.55	[19]
Teflon® AF-2400	3600	1.06	[21]

Table S4 MOF stoichiometry verification

MOF	Stoichiometry from experiments	Stoichiometry from CoRE MOF 2019 database
CIQYUX[22]	$[Co_5(OH)_2(OAc)_8](H_2O)_x$[22] or $[Co_5C_{16}H_{26}O_{18}](H_2O)_x$	$[Co_5(OH)_2(OAc)_8]_4$ or $Co_{20}C_{64}H_{104}O_{72}$
FOHRUR[23]	$[Mn_3(L)_2(bib)_2(H_2O)_4]\cdot 4H_2O$ or<br>$[Mn_3C_{54}H_{34}N_8O_{12}(H_2O)_4]\cdot 4H_2O$<br>$H_3L$ = biphenyl-3,3′,5-tricarboxylic acid, $C_{15}H_{10}O_6$<br>bib = 1,4-bis(1*H*-imidazol-4-yl)benzene, $C_{12}H_{10}N_4$	$Mn_3(L)_2(bib)_2$ or<br>$Mn_3C_{54}H_{34}N_8O_{12}$
IHOTEG[24]	$Mg_5(\mu_3\text{-}OH)_2(OAc)_8\cdot 1.19H_2O$ or<br>$Mg_5C_{16}H_{26}O_{18}\cdot 1.19H_2O$	$[Mg_5(\mu_3\text{-}OH)_2(OAc)_8]_8$ or<br>$Mg_{40}C_{128}H_{208}O_{144}$
SIZNIA[25]	$\{[Cu(L\text{–}CH_3)_2(H_2O)_2](H_2O)\}_n$ or<br>$\{[CuC_{14}H_{12}O_4N_2(H_2O)_2](H_2O)\}_n$<br>$L\text{–}CH_3^-$ = 5-methylnicotinate, $C_7H_6O_2N$	$[Cu(L\text{-}CH_3)_2]_4$ or<br>$Cu_4C_{56}H_{48}N_8O_{16}$
XALVOY[26]	$\{[Cu_2(1,2\text{-}BDC)_2(Fbtx)_2]\cdot 3H_2O\}_n$ or<br>$\{[Cu_2C_{40}H_{24}O_8N_{12}F_8]\cdot 3H_2O\}_n$<br>1,2-BDC = 1,2-benzenedicarboxylate, $C_8H_4O_4$<br>Fbtx = 1,4-bis(1,2,4-triazole-1-ylmethyl)-2,3,5,6-tetrafluorobenenze, $C_{12}H_8N_6F_4$	$[Cu_2(1,2\text{-}BDC)_2(Fbtx)_2]_2$ or<br>$Cu_4C_{80}H_{48}O_{16}N_{24}F_{16}$
**XULRIH**[27]	$Zn_3(H_2O)(L)(HPO_3)_3$ or $Zn_3C_{18}H_{18}O_{10}N_5P_3$<br>L= 2,4,5-tri(4-pyridyl)-imidazole, $C_{18}H_{13}N_5$	$[Zn_3(L)(PO_3)_3(H_2O)]_2$ or<br>$Zn_6C_{36}H_{30}O_{20}N_{10}P_6$

References

(1) Zimmerman, C. M.; Koros, W. J., Polypyrrolones for membrane gas separations. I. Structural comparison of gas transport and sorption properties. *Journal of Polymer Science Part B: Polymer Physics* **1999,** *37* (12), 1235-1249.

(2) Yang, L.; Fang, J.; Meichin, N.; Tanaka, K.; Kita, H.; Okamoto, K., Gas permeation properties of thianthrene-5,5,10,10-tetraoxide-containing polyimides. *Polymer* **2001,** *42* (5), 2021-2029.

(3) Shida, Y.; Sakaguchi, T.; Shiotsuki, M.; Sanda, F.; Freeman, B. D.; Masuda, T., Synthesis and properties of membranes of poly(diphenylacetylenes) having fluorines and hydroxyl groups. *Macromolecules* **2006,** *39* (2), 569-574.

(4) Wang, L.; Cao, Y.; Zhou, M.; Zhou, S. J.; Yuan, Q., Novel copolyimide membranes for gas separation. *Journal of Membrane Science* **2007,** *305* (1-2), 338-346.

(5) Nagel, C.; Günther-Schade, K.; Fritsch, D.; Strunskus, T.; Faupel, F., Free volume and transport properties in highly selective polymer membranes. *Macromolecules* **2002,** *35* (6), 2071-2077.

(6) Budd, P.; Msayib, K.; Tattershall, C.; Ghanem, B.; Reynolds, K.; McKeown, N.; Fritsch, D., Gas separation membranes from polymers of intrinsic microporosity. *Journal of Membrane Science* **2005,** *251* (1-2), 263-269.

(7) Mizumoto, T.; Masuda, T.; Higashimura, T., Polymerization of [o-(trimethylgermyl)phenyl] acetylene and polymer characterization. *Journal of Polymer Science Part A: Polymer Chemistry* **2003,** *31* (10), 2555-2561.

(8) Comesaña-Gándara, B.; Chen, J.; Bezzu, C. G.; Carta, M.; Rose, I.; Ferrari, M.-C.; Esposito, E.; Fuoco, A.; Jansen, J. C.; McKeown, N. B., Redefining the Robeson upper bounds for CO_2/CH_4 and CO_2/N_2 separations using a series of ultrapermeable benzotriptycene-based polymers of intrinsic microporosity. *Energy & Environmental Science* **2019,** *12* (9), 2733-2740.

(9) Li, Y.; Wang, X.; Ding, M.; Xu, J., Effects of molecular structure on the permeability and permselectivity of aromatic polyimides. *Journal of Applied Polymer Science* **1996,** *61* (5), 741-748.

(10) Maier, G., Gas Separation with Polymer Membranes. *Angewandte Chemie International Edition* **1998,** *37* (21), 2960-2974.

(11) Hamad, F.; Matsuura, T., Performance of gas separation membranes made from sulfonated brominated high molecular weight poly(2,4-dimethyl-l,6-phenyIene oxide). *Journal of Membrane Science* **2005,** *253* (1-2), 183-189.

(12) Kim, Y. H.; Kim, H. S.; Kwon, S. K., Synthesis and characterization of highly soluble and oxygen permeable new polyimides based on twisted biphenyl dianhydride and spirobifluorene diamine. *Macromolecules* **2005,** *38* (19), 7950-7956.

(13) Teraguchi, M.; Masuda, T., Poly(diphenylacetylene) membranes with high gas permeability and remarkable chiral memory. *Macromolecules* **2002,** *35* (4), 1149-1151.

(14) Swaidan, R.; Ghanem, B.; Pinnau, I., Fine-Tuned Intrinsically Ultramicroporous Polymers Redefine the Permeability/Selectivity Upper Bounds of Membrane-Based Air and Hydrogen Separations. *ACS Macro Lett* **2015,** *4* (9), 947-951.

(15) Fitch, M. W.; Koros, W. J.; Nolen, R. L.; Carnes, J. R., Permeation of several gases through elastomers, with emphasis on the deuterium/hydrogen pair. *Journal of Applied Polymer Science* **2003,** *47* (6), 1033-1046.

(16) Chiou, J. S.; Paul, D. R., Gas Permeation in a Dry Nafion Membrane. *Industrial & Engineering Chemistry Research* **1988,** *27* (11), 2161-2164.

(17) Merkel, T. C.; Pinnau, I.; Prabhakar, R.; Freeman, B. D., Gas and vapor transport properties of perfluoropolymers. *Materials science of membranes for gas and vapor separation* **2006**, 251-270.
(18) Teplyakov, V. V.; Paul, D. R.; Bespalova, N. B.; Finkelshtein, E. S., Gas Permeation in a Fluorine-Containing Polynorbornene. *Macromolecules* **1992,** *25* (16), 4218-4219.
(19) Macchione, M.; Jansen, J. C.; De Luca, G.; Tocci, E.; Longeri, M.; Drioli, E., Experimental analysis and simulation of the gas transport in dense Hyflon® AD60X membranes: Influence of residual solvent. *Polymer* **2007,** *48* (9), 2619-2635.
(20) Jansen, J.; Macchione, M.; Drioli, E., On the unusual solvent retention and the effect on the gas transport in perfluorinated Hyflon AD® membranes. *Journal of Membrane Science* **2007,** *287* (1), 132-137.
(21) Pinnau, I.; Toy, L. G., Gas and vapor transport properties of amorphous perfluorinated copolymer membranes based on 2, 2-bistrifluoromethyl-4, 5-difluoro-1, 3-dioxole/tetrafluoroethylene. *Journal of Membrane Science* **1996,** *109* (1), 125-133.
(22) Lin, Z. J.; Wragg, D. S.; Warren, J. E.; Morris, R. E., Anion control in the ionothermal synthesis of coordination polymers. *J Am Chem Soc* **2007,** *129* (34), 10334.
(23) Li, Y. P.; Chai, Y.; Yang, G. P.; Miao, H. H.; Cui, L.; Wang, Y. Y.; Shi, Q. Z., Metal ions and solvents ratio co-regulate four new magnetic coordination polymers based upon an unsymmetric tricarboxylate acid ligand. *Dalton Trans* **2014,** *43* (28), 10947-55.
(24) Scheurell, K.; Troyanov, S. I.; Kemnitz, E., Structural Chemistry of Basic Magnesium Acetates, $Mg_5(\mu_3{}^-OH)_2(OAc)_8{\cdot}nL$ and $Mg_3(\mu_4{}^-O)(OAc)_4$. *Zeitschrift für anorganische und allgemeine Chemie* **2015,** *641* (6), 1106-1109.
(25) Li, C.-P.; Chen, J.; Liu, P.-W.; Du, M., Structural diversity of 5-methylnicotinate coordination assemblies regulated by metal-ligating tendency and metal-dependent anion effect. *CrystEngComm* **2014,** *16* (28).
(26) Chen, S.-C.; Lu, S.-N.; Tian, F.; Li, N.; Qian, H.-Y.; Cui, A.-J.; He, M.-Y.; Chen, Q., Highly selective aerobic oxidation of alcohols to aldehydes over a new Cu(II)-based metal-organic framework with mixed linkers. *Catalysis Communications* **2017,** *95*, 6-11.
(27) Wang, C. M.; Chang, T. Y.; Lee, L. W.; Lin, H. M.; Lu, K. L.; Lii, K. H., The first zinc phosphite with remarkable structural and functional transformations. *Chem Commun (Camb)* **2015,** *51* (37), 7824-6.
